# Carbon Adsorbents With Dual Porosity for Efficient Removal of Uremic Toxins and Cytokines from Human Plasma

**DOI:** 10.1038/s41598-017-15116-y

**Published:** 2017-11-02

**Authors:** D. Pavlenko, D. Giasafaki, G. Charalambopoulou, E. van Geffen, K. G. F. Gerritsen, T. Steriotis, D. Stamatialis

**Affiliations:** 1 (Bio)artificial organs, Department of Biomaterials Science and Technology, MIRA Institute for Biomedical Engineering and Technical Medicine, University of Twente, P.O. Box 217, 7500 AE Enschede, The Netherlands; 2 National Center for Scientific Research “Demokritos”, Agia Paraskevi Attikis, 15341 Athens, Greece; 3 Department of Nephrology and Hypertension, University Medical Centre Utrecht, P.O. Box 85500, 3508 GA Utrecht, The Netherlands

## Abstract

The number of patients with chronic kidney disease increases while the number of available donor organs stays at approximately the same level. Unavoidable accumulation of the uremic toxins and cytokines for these patients comes as the result of malfunctioning kidneys and their high levels in the blood result in high morbidity and mortality. Unfortunately, the existing methods, like hemodialysis and hemofiltration, provide only partial removal of uremic toxins and/or cytokines from patients’ blood. Consequently, there is an increasing need for the development of the extracorporeal treatments which will enable removal of broad spectrum of uremic toxins that are usually removed by healthy kidneys. Therefore, in this work we developed and tested ordered mesoporous carbons as new sorbents with dual porosity (micro/meso) that provide selective and efficient removal of a broad range of uremic toxins from human plasma. The new sorbents, CMK-3 are developed by nanocasting methods and have two distinct pore domains, i.e. micropores and mesopores, therefore show high adsorption capacity towards small water soluble toxins (creatinine), protein-bound molecules (indoxyl sulfate and hippuric acid), middle molecules (β-2-microglobulin) and cytokines of different size (IL-6 and IL-8). Our results show that small amounts of CMK-3 could provide selective and complete blood purification.

## Introduction

Patients with kidney malfunction suffer from consequences of unavoidable accumulation of uremic toxins in their blood. All these uremic toxins are generally divided into three main groups based on their size and/or properties. The first group consists of small water soluble molecules with molecular weights (MW) lower than 500 Da. Molecules from this group, like creatinine and urea, are traditionally removed well by diffusion-based membrane treatment, for example hemodialysis, using “low-flux” dialysis membranes. The second group of toxins consists of “middle molecules’, which have MW larger than 500 Da, for example β-2-microglobulin (β_2m_) and cytokines like IL-6 and IL-8, and as such they have low diffusion rates. Removal of these molecules can be improved by using “high-flux” membranes with more open structure (in comparison to “low flux” membranes) and by adding convection (hemofiltration) to diffusion (a treatment called hemodiafiltration)^1^. The third group of uremic toxins comprises of solutes that are bound to human serum albumin (HSA) in human blood (protein-bound toxins, PBT). As both high-flux and low-flux dialysis membranes are designed to retain albumin, only the free fraction of these toxins (unbound to albumin) is removed by hemodialysis, hemofiltration and hemodiafiltration^1,2^.

Adsorption techniques can in principle offer better removal of PBT, middle molecules and cytokines from patients’ blood. For example, during hemoperfusion the patients’ blood is pumped through a column packed with adsorbent particles. These particles provide rather fast and efficient adsorptive removal of all the toxins they are designed to remove. As the result this treatment found its application in intensive medical care units for removal of inflammatory cytokine mediators in septic patients. However, hemoperfusion does not offer control over the fluid balance and ability to remove urea. Nevertheless, combination of both modes of blood purification, membrane- and adsorption-based, can in principle provide better blood purification to kidney patients. For example, clinically relevant blood purification methods like Adsorbents Recirculation System (MARS, Gambro), Prometheus System (Fresenius Medical Care) and Coupled Plasma Plasma Filtration and Adsorption (Bellco) successfully use both adsorption and membrane-based techniques to remove the toxins from patients^3^. Besides, recently our laboratory successfully combined the benefits of adsorption and diffusion in one membrane, the mixed matrix membrane (MMM), which consists of two layers: a selective inner layer which is responsible for blood contact and selectivity and an outer layer where adsorptive particles are incorporated in a highly porous membrane matrix^4,5^. The addition of the outer layer with adsorptive particles improves the concentration gradients of the toxins across the membrane resulting in high removal of PBT *in vitro* in comparison to current dialysis membranes^4,6^.

In all the above cases the properties of adsorptive particles are very important for successful therapy. Therefore, significant efforts have been focused on the development of appropriate adsorbent materials which can remove a broad range of blood toxins from patients’ blood. For example, Harm *et al*.^3^ showed that the pore size of the sorbent material determines the selectivity towards the blood toxins. So, the efficiency of the adsorbent can be adjusted to the needs of the treatment by tailoring its pore size distribution. In fact, microporous adsorbent particles (with pores < 2 nm according to IUPAC) cannot remove substances with high and middle Mw. Larger molecules, such as β_2m_, and cytokines, such as IL-6, IL-8, and most of PBTs could be better removed by mesoporous sorbents (pores of 2–50 nm) due to improved pore accessibility. Based on this, various researchers focused on development of adsorbent materials with hierarchical porosity: sorbents with pores of two or more length scales^6^ (micro-, meso- and macro pores). The first results of, for example, Presser *et al*. were quite encouraging^7^, however authors indicated that material performance, e.g. selectivity, can be further improved by narrowing the pore size distribution of the sorbents. Besides, one limitation of these sorbents was the high adsorption of large protein molecules (like HSA and often indicated by protein loss), which limited the accessibility of small pores and, thus, reduce the adsorptive capacity of the material^7^.

In this work, we hypothesize that carbon-based sorbent materials with two well-defined pores sizes could provide better removal of a broad range of the uremic toxins and cytokines. Therefore, we use an ordered nanoporous sorbent material (CMK-3 type) which has two distinct pore domains, i.e. micropores and mesopores. This sorbent consists of a set of parallel rods that are hexagonally packed and interconnected with thin carbon strands. Due to the carbon precursor pyrolysis process these rods are microporous (0.8–1 nm), and are responsible for the efficient adsorption of small water soluble molecules and PBTs. The space between the rods creates the well-defined and easily tuneable mesoporous system (5 nm in our case) that should achieve the removal of middle molecules and cytokines without blocking the micropores. For the proof of concept here, we investigated the removal of a broad range of toxins from human plasma by CMK-3 type adsorbent, including small water soluble molecules, like creatinine (113 Da), middle molecules, like β_2m_ (11.6 kDa), and PBTs like hippuric acid (179 Da, 48% bound to HSA) and indoxyl sulfate (213 Da, 98% bound to HSA), as well as the removal of two cytokines, IL-6 and IL-8 (24 kDa and 8 kDa respectively). The performance of CMK-3 was compared with two commercially available carbon-based sorbents with predominant mesoporosity (Norit A Supra, 3 nm) and microporosity (Takeda 5 A, 0.6 nm). Norit A Supra was previously used by our laboratory in MMM and showed good ability to remove creatinine and PBTs from human plasma, while Takeda was used practically as a negative control.

## Results and Discussion

### Characterization of synthesized carbons

**Figure 1:**
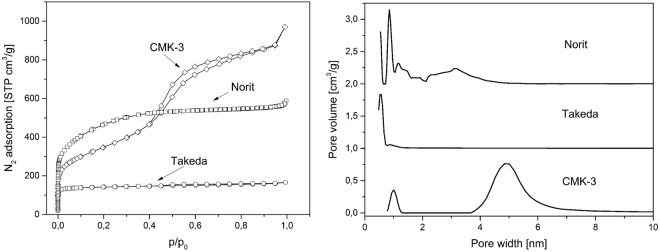
N_2_ adsorption-desorption isotherm for studied carbon materials (left) and Pore size distribution for studied carbons obtained from quenched solid state functional theory (right).

In this study the performance of CMK-3 was compared with two commercially available carbon-based sorbents: Norit A Supra that consists of micropores (<0.7 nm, i.e. 0.5 nm), micropores of about 0.9 nm and small mesopores of around 3 nm and Takeda 5 A which is clearly an ultra-microporous material with pores of 0.6 nm (<0.7 nm). As for the CMK-3, it is a micro-mesoporous material with mesopores of ~5 nm and micropores of ~1 nm (Fig. 1) in accordance with literature data^8^. Additionally, there is a difference in the Brunauer – Emmett – Teller (BET) surface area between the carbon materials studies. Here, the specific surface area of CMK-3 is around 1250 m^2^/g, higher than the one of Takeda 5 A (560 m^2^/g), but lower than the one of Norit A Supra (approximately 1700 m^2^/g).

Sorbent properties are described in Table 1. Both CMK-3 and Norit A Supra have relatively small diameter, 1–15 µm and 2–40 µm, respectively. The Takeda particles have noticeably higher particle size range (3–150 µm) with predominantly big particles (>100 µm) and rather low surface area (500–600 m^2^/g). The Norit particles have the highest surface area of all materials.

**Table 1:** Material properties of studied nanoporous carbons.

	Particle size *(µm)*	Pore size *(nm)*	BET surface area *(m* ^2^ */g)*
CMK-3	1–15	0.8–1 and 5	1250
Takeda	3–150	0.6	560
Norit A Supra	2–40	0.9 and 3	1700

### Adsorption of total plasma proteins

**Figure 2:**
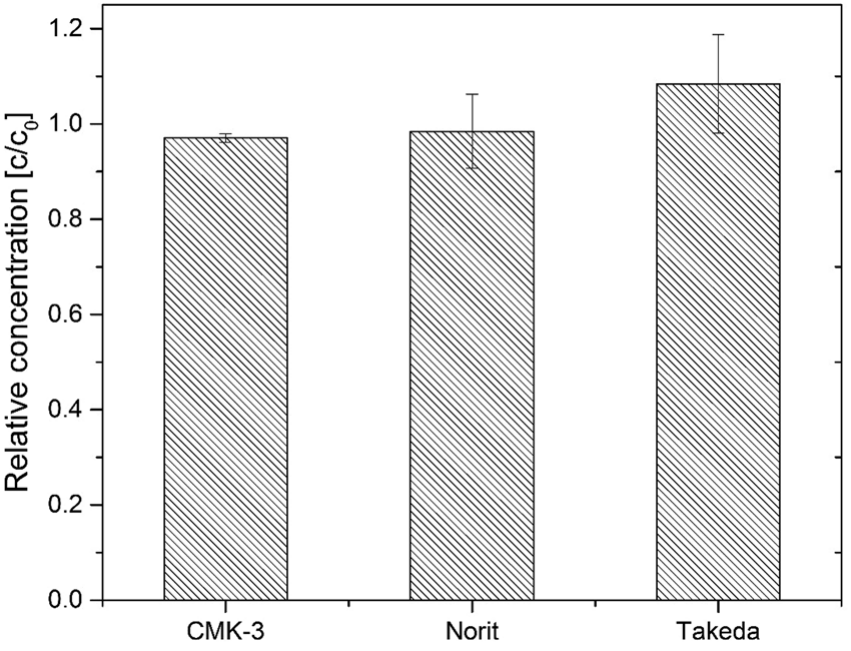
Total plasma protein levels after the adsorption experiments.

Figure 2 presents the relative total plasma protein concentration after the contact with nanoporous carbons for 4 hours. All data were normalized to a control (plasma without any particles). No significant difference (p < 0.05, n = 3) between initial (103.5 g/l) and final protein concentrations was observed for all used carbon materials.

In other studies^3,9^ where carbon materials were designed to remove cytokines from human plasma, researchers observed noticeable plasma protein adsorption to the particles. Howel *et al*.^10^ also reported that dextran coating of the particle could reduce HSA adsorption, though not completely. Even though HSA plays important role in body hemostasis, negative effect of its adsorption have not yet being reported^10^. Nevertheless, albumin adsorption onto the porous materials can generally reduce the specific surface area (SSA) of the adsorbent^10^, which is expected to have negative effect on its adsorption capacity^7^. In other words, adsorption of the HSA on the carbon adsorbers would perhaps not cause any side effects, but it should be avoided to prevent that it decreases the adsorption capacity, and thus to performance, of the sorbent concerning the removal of uremic solutes. As shown in Fig. 2 the CMK-3 and the other sorbents tested here showed no significant protein adsorption.

### Adsorption of small and protein-bound toxins

**Figure 3:**
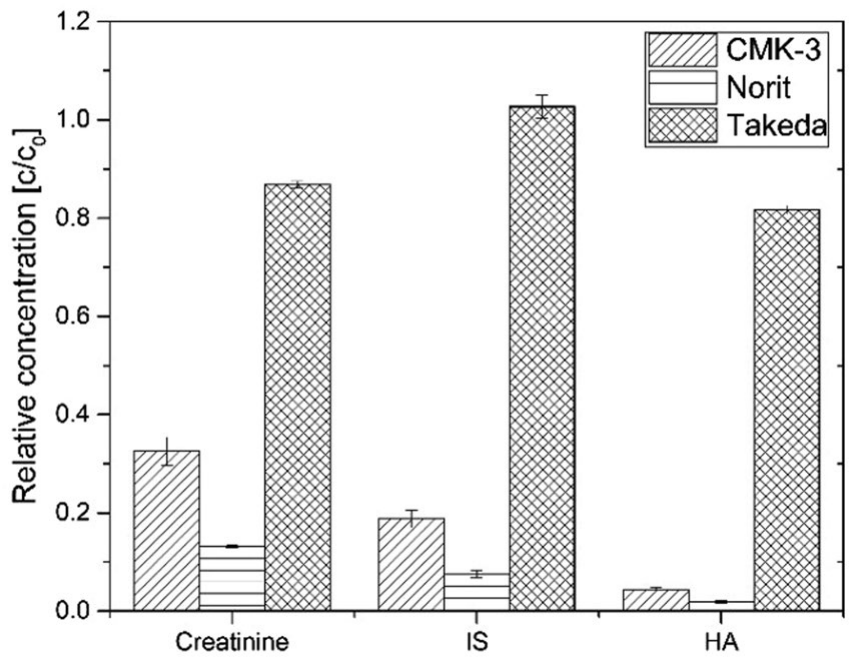
Relative plasma concentrations of small water soluble and protein-bound toxins after contact with carbon materials for 4 hours.

Figure 3 shows the average relative concentrations of creatinine, HA and IS in human plasma (normalized by the initial toxin concentration) after contact with the tested adsorbents. First, creatinine, marker molecule of kidney function, belongs to the group of small water soluble molecules; its adsorptive removal is mainly driven by its diffusion to the adsorptive sites of the nanoporous materials and sorbent affinity to creatinine itself. From Fig. 3 it is clear that the CMK-3 and Norit adsorb the majority of the creatinine from the plasma, while Takeda particles only achieve minor adsorption. The small difference in the performance between CMK-3 and Norit can be attributed to difference in their SSA rather than their affinities to creatinine. In fact, when we normalized the amount of toxins adsorbed to SSA (see Table 2), we found that CMK-3 actually adsorbs slightly higher amount in comparison to Norit (14.0 and 10.7 mg/1000 m^2^ respectively). Based on these data, to remove the daily creatinine production of kidney patients (around 1800 mg), one would need only 128 g of CMK-3. Of course, the amount of CMK-3 required could be further decreased by increasing its SSA and/or in number of pores per gram of material. This can be rather easily achieved, e.g. by using less carbon precursor per gram of SBA-15 silica or with mild activation (heating under CO_2_ flow).

**Table 2:** Removal of all studied molecules normalized for the gram and SSA of the tested materials.

	Small water soluble (MW)		Protein-bound solutes (MW)				Cytokines (MW)			
	Creatinine (113 Da)		IS (213 Da)		HA (179 Da)		IL-6 (24 kDa)		IL-8 (8 kDa)	
	*mg/g*	*mg/1000* *m* ^2^	*mg/g*	*mg/1000* *m* ^2^	*mg/g*	*mg/1000* *m* ^2^	*ng/g*	*ng/1000* *m* ^2^	*ng/g*	*ng/1000* *m* ^2^
CMK-3	14.0 ± 2.3	11.2 + 1.9	3.2 ± 1.4	2.6 ± 1.2	12.2 ± 0.3	9.8 ± 0.3	32.9 ± 7.1	26.4 ± 5.7	80**	64**
Norit	18.1 ± 0.2	14.5 ± 0.1	3.7 ± 0.6	2.9 ± 0.4	12.6 ± 0.2	10.1 ± 0.1	0*	0*	72.6 ± 1.8	58.0 ± 1.4
Takeda	2.8 ± 0.6	2.2 ± 0.5	0*	0*	2.3 ± 0.7	1.9 ± 0.5	0*	0*	13.1 ± 5.4	10.5 ± 4.4

*Value below detection limits.

**Indicated that the saturation of the carbon material was not reached as all the IL-8 was removed from the plasma solution.

Figure 3 also depicts the removal of HA and IS by the tested materials from human plasma samples. The IS removal is slightly lower than the corresponding one for HA. Even though the studied PBT had different initial concentration, which might also have influenced the final result, we could attribute the difference in the removal to different albumin binding properties of the two studied uremic toxins. Similar to creatinine removal, the amount of PBT/m^2^ adsorbed by CMK-3 is slightly higher than Norit (see Table 2). Based on the adsorption results for IS and HA by CMK-3, one only needs 22 g of CMK-3 material to remove the mean daily excretion of the IS and HA by a patient (69 and 270 mg^11^, respectively).

Generally, we find that the degree of binding to albumin of respective toxins have direct correlation to their removal and is consistent to other literature^12^. The higher the concentration of the free fraction in plasma, the higher the removal. However, due to usually high protein binding properties most of the PBTs are poorly removed by dialysis^1,13,14^. Unlike hemodialysis, carbon materials are usually in close contact with blood or plasma, making the diffusion length for toxin free fraction much smaller and, thus, making adsorption a good alternative and/or complement to conventional hemodialysis^3–5,15,16^.

### Adsorption of middle molecules and cytokines

**Figure 4:**
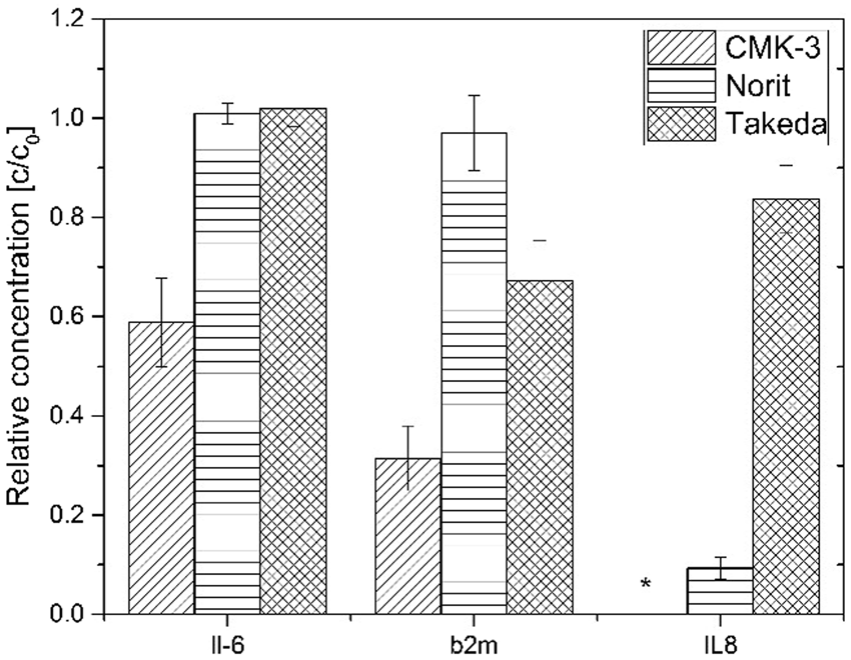
Relative plasma concentrations of middle molecules and cytokines toxins after contact with carbon materials for 4 hours.

Figure 4 compares the removal of the β_2m_ and of IL-6 and IL-8 from human plasma after contact with the studied sorbents. IL-8, the smallest cytokine (MW = 8 kDa) is completely adsorbed by the CMK-3 in all samples after 4 hours. The concentration of the IL-8 was also decreased by 90% by the Norit sorbent whereas Takeda sorbent performed very modestly by decreasing the IL-8 concentrations only by 16%.

The CMK-3 carbon also removed 42% of IL-6 (24 kDa) and 68% of the β_2m_ (11.8 kDa) after 4 hours of adsorption while both Norit and Takeda performed rather poorly (even though Takeda removed 22% of the β_2m_ from human plasma samples). The main reason for the high removal of the cytokines and middle molecules is the porous structure of CMK-3. It seems that the distance between the rods (5 nm) that creates the mesoporosity is suitable for the adsorption of the selected cytokines without the HSA adsorption.

Direct comparison of our results to other literature studies is not straightforward due to differences in testing protocols (different starting concentrations, experimental time, plasma-to-carbon ratios etc.) used. There are a few studies, however, where comparison is possible. For example, Song *et al*.^17^ could remove 12.5 ng/g of IL-6 by using commercially available Cytosorb^TM^ sorbent, which is much lower than the removal of the 33 ng/g by CMK-3. Their experiments were carried out in dynamic conditions and showed fast adsorption of IL-6. Besides, in contrast to our work where we found no protein loss; in their study the total protein levels decreased rapidly during the first hour of the experiment indicating the low selectivity of the Cytosorb^TM^ materials. Another study by Howel and co-workers^18^ used adsorptive beads that have slightly higher removal of IL-6 than Cytosorb^TM^, with 14.3 ng/g of IL-6 removed, which is still much lower in comparison to CMK-3 carbon materials with removal of 33 ng/g of IL-6. Other studies showed that the adsorption of the cytokines is significantly influenced by the number and size of mesopores present in the adsorbent material^19,20^. There, the tested adsorbing material, carbide-derived carbon, was able to remove 1.25 ng/g of IL-8 and 5 ng/g of IL-6. In our work, CMK-3 removes much more, namely 80 ng/g of IL-8 and 33 ng/g of IL-6. Despite the differences in testing conditions between the two studies, especially experimental time (2 hours^19^ vs 4 hours in our study) and in carbon-plasma ratios (0.2 g in 0.5 ml of plasma there, 25 mg per 4 ml of plasma, in our study) these results clearly show the great potential of CMK-3 particles for removing a broad range of toxins.

The CMK-3 particles have relatively modest surface area per gram of the material (1250 m^2^/g), which can be significantly increased, leading to even small amounts of particles necessary. Their size is small and they are not suitable as fillers to the adsorption columns, since relatively small sizes of the adsorbents there usually result in significant pressure drops^21^, and there is always increased chance of small particle leakage into the blood stream, unless a particle filter is introduced^7^. We foresee the incorporation of CMK-3 into the mixed matrix membranes (MMM) where, as we have shown earlier^21^, application of small particles does not result in noticeable pressure drops and makes it possible to combine benefits of membrane filtration and adsorption in one device^4,5,22^.

### Conclusions and Outlook

A sorbent particles, namely CMK-3, were developed with the capacity to remove high spectrum of uraemic toxins from human plasma solutions: small water soluble molecules, middle molecules and protein-bound toxins. Additionally, this material did not show tendency to lower total plasma protein levels through 4 hours of direct plasma-sorbent contact and shows potential to be used in extracorporeal blood purification treatments.

Future work of our lab will focus on incorporation of CMK-3 inside mixed matrix membranes: earlier results with incorporation of Norit A Supra (used as the reference in this study) particles inside the MMM were very promising. Results with Norit A highlighted that the MMM can increase the removal of indoxyl sulfate and p-cresyl sulfate up to 100–200% in comparison to particle-free industrial membranes^4^. As it was shown in the current study, Norit carbons perform rather well in removing the protein-bound and small water soluble molecules, but show noticeably lower performance when applied for the removal of larger cytokines and β_2m_. We believe that incorporation of the CMK-3 carbons inside MMM will not only broaden the application of the new membrane material, but will also help to avoid complications caused by relatively small adsorbent particles inside the adsorption columns, e.g. pressure drops and leakage of the particles.

## Methods

### Synthesis of mesoporous carbon CMK-3

The synthesis of the CMK-3 type ordered mesoporous carbon was carried out following the hard-templating procedure, described in details elsewhere^23^. In short, SBA-15 hexagonally ordered mesoporous silica template (Claytec Inc., USA) was infiltrated twice with an aqueous sucrose solution containing minute amounts of H_2_SO_4_. The composite was heat treated in air (6 h at 100 °C followed by 6 h at 160 °C) and consequently carbonized at 900 °C for 2 h (10 °C/min) in a temperature-programmed horizontal tubular furnace (MTI GSL-1100X) under N_2_ flow (80 ml/min). Finally, the carbon/silica composite was treated with HF (48 wt%) at room temperature to remove the silica part. In this respect, the final porous carbon is actually a negative replica of the silicious structure and thus its mesoporosity is determined by the pore size and wall thickness of the starting SBA-15 material. The carbon precursor (sucrose) pyrolysis process always creates a microporous network due to inefficient stacking of the developed graphitic platelets. Microporosity can be controlled through the pyrolysis conditions (e.g. temperature, time) and can be further increased by activation (i.e. partial oxidation). It should be noted that in our case a rather typical protocol was followed (as a proof of principle) and thus both microporosity and mesopore size and thus adsorptive capacity can certainly be optimized in the future. Details on the surface chemistry of CMK-3 can be found elsewhere^24^. In brief, the sample contains a significant amount of surface oxygen functionalities, the majority being C-O-C (ether/epoxy) groups, while smaller amounts of hydroxyl, carbonyl and carboxyl groups are also present.

### Carbon characterization

The properties of the porous carbon materials, CMK-3, Norit A Supra and Takeda, in this study were estimated via N_2_ adsorption-desorption isotherms (Autosorb-1 MP, Quantachrome). All the samples were degassed under vacuum (10^−6^ mbar) for around 12 hours at 250 °C before each measurement. Moreover, small angle x-ray scattering (SAXS) measurements were performed for the CMK-3 sample in order to verify its pore ordering.

### Static batch adsorption experiments

Healthy human plasma was obtained from Sanquin (Deventer, Netherlands) and stored frozen at −20 °C. Prior to the experiments, the plasma was defrosted at 37 °C and spiked with uraemic concentrations of small water soluble and protein bound toxins, middle molecules and cytokines, as it is shown in Table 3.

**Table 3:** Initial concentrations of the toxins and cytokines used in this study.

	MW, kDa	Concentration	Batch	Protein bounding
Creatinine	0.113	130 µg/ml	I	No
Indoxyl Sulfate	0.213	25 µg/ml	I	97.70%^12^
Hippuric acid	0.179	80 µg/ml	I	48.30%^12^
β_2m_	11.6	84.7 µg/ml	II	No
IL-6	24	500 pg/ml	II	No
IL-8	8	500 pg/ml	II	No

The blood plasma adsorption experiments were divided into two batches. First batch (batch I) was spiked with creatinine, IS and HA, while batch II was spiked with β_2m_, IL-6 and IL-8. In all experiments 25 mg of carbon material was added to 4 ml of human plasma. The obtained solutions were shaken gently and left in an incubator at 37^o^C for 4 hours. After adsorption, all samples (triplicates for each measurement) were centrifuged at 3500 rpm for 10 minutes to separate the adsorbents from plasma. Afterwards, the concentrations of uremic toxins and cytokines were analysed as described below.

### Total protein and uremic toxin analysis

The β_2m_ concentration analysis was performed with β_2m_ ELISA kit (Siemens, Germany). The concentrations of the cytokines were identified by specific ELISA kits (BioLegend, Inc, Germany). The creatinine concentrations in plasma were determined with Creatinine Assay Kit from Sigma-Aldrich (Netherlands). The total protein concentrations were analysed by the Protein Assay kit (BioRad Laboratories GmbH).

For the analysis of plasma levels of PBTs, the plasma samples were deproteinized by heat treatment and filtered through 30 kDa filters (Amicon Ultracel-30 K, Merck Millipore Ltd). Subsequently, the concentrations of IS and HA were analysed following the protocol described by Meert *et al*.^13^.

### Statistics

All results are presented as average values with corresponding standard deviations (n = 3). Software package IBM SPSS Statistics was used for comparative statistical analysis (Independent student t-test, p < 0.05).
